# Umbilical cord blood infusion in the treatment of aplastic anemia: A single center prospective study

**DOI:** 10.1038/s41598-025-18188-3

**Published:** 2025-10-03

**Authors:** Xianghui Liu, Zhirui Zhao, Weiwei Zhu, Tianjie Han, Lijun Li, Xin Li, Yue Ma, Zhe Yu

**Affiliations:** Department of Hematology, Shandong Provincial Third Hospital, Shandong University, Jinan, 250013 Shandong China

**Keywords:** Umbilical cord blood, Aplastic anemia, Cyclosporine, Hetrombopag, Cell therapy, Stem-cell research, Anaemia

## Abstract

The current standard first-line therapy for aplastic anemia (AA) consists of antithymocyte globulin (ATG), cyclosporine, and thrombopoietin receptor agonists (TPO-RAs). A subset of patients exhibit treatment intolerance or develop refractory/relapsed disease, for whom treatment options are limited. Umbilical cord blood exhibits immunomodulatory effects, regulates the bone marrow microenvironment, and promotes hematopoietic reconstitution, thereby demonstrating therapeutic potential for AA. In this prospective clinical study, cord blood infusion was administered in combination with cyclosporine and hetrombopag for the treatment of AA (n = 11). The primary endpoint was the hematologic response at 3 months post-treatment. By 3 months, 8 of 11 patients achieved at least one lineage hematologic response. By 6 months, 3 patients attained complete response (CR), 5 achieved partial response (PR), yielding an overall response rate (ORR) of 8/11. After a median follow-up of 23 months, 5 patients maintained sustained CR and 3 sustained PR, with the ORR remaining at 8/11. The median time to trilineage response was 112 days (range: 18–168 days) among 8 responsive patients. Two patients with SAA refractory to treatment succumbed to pulmonary infections and pneumorrhagia. No significant adverse reactions were observed in cord blood-treated patients. This small-scale study introduces a novel regimen combining umbilical cord blood infusion, cyclosporine, and hetrombopag for treating aplastic anemia. With a median follow-up of 23 months, the overall response rate reached 8/11 and the CR rate 5/11. The safety and efficacy of this regimen were preliminarily assessed, suggesting a potential therapeutic alternative for patients ineligible for standard immunosuppressive therapy.

## Introduction

Aplastic anemia (AA) is a hematopoietic stem cell disorder characterized by pancytopenia and hypocellular bone marrow. The pathogenesis is postulated to result from cytotoxic T lymphocyte-mediated depletion of hematopoietic stem cells^1^. Although allogeneic hematopoietic stem cell transplantation (HSCT) offers potential cure, immunosuppressive therapy (IST) combining anti-thymocyte globulin (ATG) and cyclosporine remains an effective first-line alternative, achieving initial response rates of 60–70%. However, 30–60% of patients ultimately experience disease relapse^1–3^. The introduction of thrombopoietin receptor agonists (TPO-RAs) such as eltrombopag has improved response rates, yet approximately 20% of patients develop refractory AA and 30–40% experience relapse after initial response^1,2,4^.

Refractory patients with persistent pancytopenia remain at high risk for severe infections or hemorrhagic complications. However, immunosuppression-based second-line therapies have not substantially improved response rates. Previous therapeutic investigations predominantly employed strategies to intensify immunosuppressive efficacy, including the adoption of alemtuzumab^5^, cyclophosphamide^6–8^ and rabbit ATG^9^, and adjunctive sirolimus^10^ or mycophenolate mofetil^11^. These regimens failed to demonstrate improved clinical outcomes while incurring additional toxicities. Furthermore, earlier investigations incorporating granulocyte-stimulating factors, erythropoiesis-stimulating agents, or androgens similarly showed no significant clinical benefits^12^. Therefore, there remains an unmet clinical need for novel therapeutic approaches beyond HSCT for AA management.

In 2021, hetrombopag, a TPO receptor agonist, was approved in China as second-line therapy for AA^13^. Clinical trial data showed that 23 of 55 (41.8%) relapsed/refractory patients achieved at least one lineage hematologic response by week 18 with hetrombopag monotherapy^14^. Umbilical cord blood contains abundant hematopoietic stem cells, NK cells, and naive T lymphocytes, with its enriched regulatory T cell subsets and other cellular components potentially serving as immunomodulators to regulate the bone marrow microenvironment^15^. Clinical investigations have employed cord blood as third-party donor cells in hematopoietic stem cell transplantation^16,17^ and as consolidation therapy for elderly acute myeloid leukemia^18^. Furthermore, cord blood has been extensively explored in oncology^19^, regenerative medicine^20^, and autoimmune disorders^21^. The immunomodulatory properties and regenerative capacity of cord blood-derived cells suggest therapeutic potential across multiple diseases. Herein, we investigate its novel application in aplastic anemia management.

In our study, cord blood infusion was combined with cyclosporine and hetrombopag to treat AA patients.

### Patients

From August 2020 to February 2024, eleven AA patients were enrolled, including 6 with SAA and 5 with non-SAA. All patients met clinical indications for therapeutic intervention: SAA, transfusion-dependent non-SAA, or neutropenic non-SAA. The pre-treatment disease duration ranged from 1 to 62 months (median: 1 month). Only two patients had prior failed IST. Among the nine IST-untreated patients, four were ineligible due to advanced age (> 65 years); three had contraindications from active comorbidities (pulmonary/urinary infections or gastrointestinal hemorrhage); and two could not access IST because of financial constraints. No patients had prior hematopoietic stem cell transplantation. Among four patients aged < 40 years, HSCT was precluded in all cases because of uncontrolled infections (n = 2) or financial inaccessibility (n = 2). Prior to enrollment, bone marrow aspiration and biopsy were conducted to rule out myelodysplastic syndrome (MDS) through morphological, cytogenetic and molecular genetic examination. Flow cytometry was utilized to evaluate paroxysmal nocturnal hemoglobinuria (PNH), quantifying the proportion of red blood cells and neutrophils deficient in glycosylphosphatidylinositol (GPI)-anchored proteins. A PNH clone was defined as present if this deficiency exceeded 1% in either cell lineage. All patients had Eastern Cooperative Oncology Group (ECOG) performance status scores of 0–2 and showed no significant cardiac, hepatic, or renal dysfunction.

### Treatment protocol

Enrolled patients received combined therapy with cord blood, cyclosporine, and hetrombopag. Cord blood was administered via intravenous infusion at 15-day intervals. The cord blood unit per infusion contained > 7 × 10⁸ mononuclear cells (MNCs) and > 5 × 10⁷ CD34⁺ cells. Pharmacological dosing was as follows: cyclosporine at 3–5 mg/kg/day with trough concentration maintained between 150–250 μg/L; hetrombopag initiated at 7.5 mg daily, with subsequent dose adjustments based on platelet counts to maintain the minimal effective dose sustaining therapeutic response. G-CSF was administered when the patient’s ANC was < 0.5 × 10⁹/L and discontinued once the ANC rised by more than 0.5 × 10⁹/L.

### Response assessment

The primary endpoint was defined as hematologic response at 3 months post-treatment, assessed through improvements in absolute neutrophil count, hemoglobin level, and platelet count. Criteria for hematologic response are detailed in the Table 1. Hematologic improvements attributed to exogenous growth factors or blood product transfusions were not considered treatment responses. Specifically, efficacy evaluations following granulocyte colony-stimulating factor (G-CSF) administration or blood product transfusions required a minimum 2-week washout period prior to assessment. Indications for component transfusion: HGB ≤ 60 g/L, PLT ≤ 10 × 10⁹/L, or PLT ≤ 20 × 10⁹/L with clinically significant bleeding tendency. Transfusion dependence is defined as requiring at least one component transfusion every 2 months for ≥ 4 months. For RBC transfusion-dependent patients, the response criterion is ≥ 4-unit reduction in transfusion requirement per 2-month period. For platelet transfusion-dependent patients, evaluate platelet count and assess response at least 7 days post-transfusion.

**Table 1:** Criteria for hematologic response.

Neutrophil Response	• If baseline ANC below 0.5 × 10^9^/L, increase in ANC by ≥ 100% • If baseline ANC between 0.5–1.5 × 10^9^/L, increase in ANC by ≥ 0.5 × 10^9^/L
Erythroid Response	• A ≥ 4-unit reduction in transfusion requirement per 2-month period in transfusion-dependent patients • Increase in hemoglobin by ≥ 15 g/L in transfusion-independent patients
Platelet Response	• If baseline platelet count < 10 × 10^9^/L, increase in platelet count by ≥ 100% and increase to over 10 × 10^9^/L • If baseline platelet count ≥ 10 × 10^9^L, increase in platelet count by ≥ 20 × 10^9^/L

Abbreviation: ANC, absolute neutrophil count.

Secondary endpoints included response at 6 months post-treatment, time to response onset, and duration of response. Additionally, patient response status was evaluated according to established efficacy criteria^22^, with documentation of response duration.

### Statistics

Continuous variables were expressed as median with range. Set relationships among hematologic responses were visualized with Venn diagrams. Longitudinal therapeutic response patterns were depicted with swimmer plots. Pre- versus post-treatment trilineage counts were compared with Wilcoxon signed-rank test. Statistical analyses were conducted using SPSS 26.0 (IBM Corp.).

## Results

### Baseline characteristics

From August 2020 to February 2024, eleven AA patients were enrolled, with a median follow-up duration of 23 months (range: 3–47 months). Detailed demographic and clinical characteristics are summarized in Table 2.

**Table 2:** Characteristics of the Patients at Baseline.

Characteristic	N = 11
Male/Female (n)	6/5
Age (years), median (range)	57(9–84)
Disease duration, median (range)	1(1–62)
Severity SAA (n) Non-SAA (n)	6 5
Previous treatment regimens Cyclosporine ATG + cyclosporine	9 2
Baseline laboratory value, median (range) ANC (10^9^/L) Hemoglobin (g/L) Reticulocyte (10^9^/L) Platelet (10^9^/L)	0.94 (0.17–3.58) 61 (44–104) 1.7 (0.1–98.5) 24 (5–105)
PNH clones (n) Yes No	2 9
Chromosomal karyotype abnormality (n) + 8	1
MDS related mutation (n)	1
Number of cord blood infusions One (n) Two (n) Three (n) Four (n) Five (n)	3 2 3 2 1

Abbreviation: ANC, absolute neutrophil count; MDS, myelodysplastic syndrome; HSCT, hematopoietic stem cell transplantation; HLA, human leukocyte antigen.

### Cord blood infusions

Eleven patients received 1–5 cord blood infusions (median of 3 infusions) administered at 15-day intervals. Among 28 total infusions, the median infused total nucleated cells (TNCs) were 19.3 × 10^8^ (range: 11.3–30.4 × 10^8^), with a median CD34 + cell dose of 7.0 × 10^6^ (range: 2.6–21.6 × 10^6^). Given that each umbilical cord blood unit was sourced from a distinct newborn, resulting in variable cell counts, and patients received infusions from a single unit, only ABO-compatible units exhibiting relatively higher cell counts could be selected. This resulted in inter-patient variability in the administered cell dose.

### Hematologic response

As shown in Table 3, eight patients maintained sustained trilineage hematologic responses following cord blood infusion at the final follow-up among 11 enrolled patients, while three patients failed to achieve response in any lineage. Pre- and post-treatment trilineage counts were shown in Table 4. Platelet counts showed statistically significant increases at 6 months post-treatment, as did hemoglobin levels. Neutrophil counts achieved statistical significance by the end of follow-up.

**Table 3:** Hematologic response of eleven AA patients.

No	Age	Diagnosis	Response	CR/PR	Time to trilineage response (days)	Duration of trilineage response (months)	Follow-up (months)	Survival
1	70	SAA	NR	NR	/	/	5	Death
2	42	SAA	Trilineage	PR	37	6	7	Survival
3	60	Non-SAA	NR	NR	/	/	7	Survival
4	67	SAA	Trilineage	CR	168	21	27	Survival
5	57	Non-SAA	Trilineage	CR	142	12	17	Survival
6	84	SAA	Trilineage	CR	161	41	46	Survival
7	74	SAA	NR	NR	/	/	3	Death
8	9	Non-SAA	Trilineage	CR	87	44	47	Survival
9	21	Non-SAA	Trilineage	PR	81	20	23	Survival
10	28	Non-SAA	Trilineage	PR	137	26	30	Survival
11	34	SAA	Trilineage	CR	18	37	37	Survival

Abbreviation: SAA, severe aplastic anemia; NR, none response; PR, partial response; CR, complete response.

**Table 4:** Pre- and post-treatment trilineage counts (n = 11).

	ANC (median)	Hemoglobin (median)	Platelet (median)
Baseline (n = 11)	0.94	61	24
3-month (n = 11)	1.44 P = 0.328	70 P = 0.213	33 P = 0.062
6-month (n = 9)	2.03 P = 0.123	91 P = 0.021	70 P = 0.008
12-month (n = 7)	1.99 P = 0.128	122 P = 0.018	110 P = 0.018
Final follow-up (n = 11)	2.9 P = 0.008	128 P = 0.016	121 P = 0.01

Abbreviation:ANC, absolute neutrophil count.

**Fig. 1:**
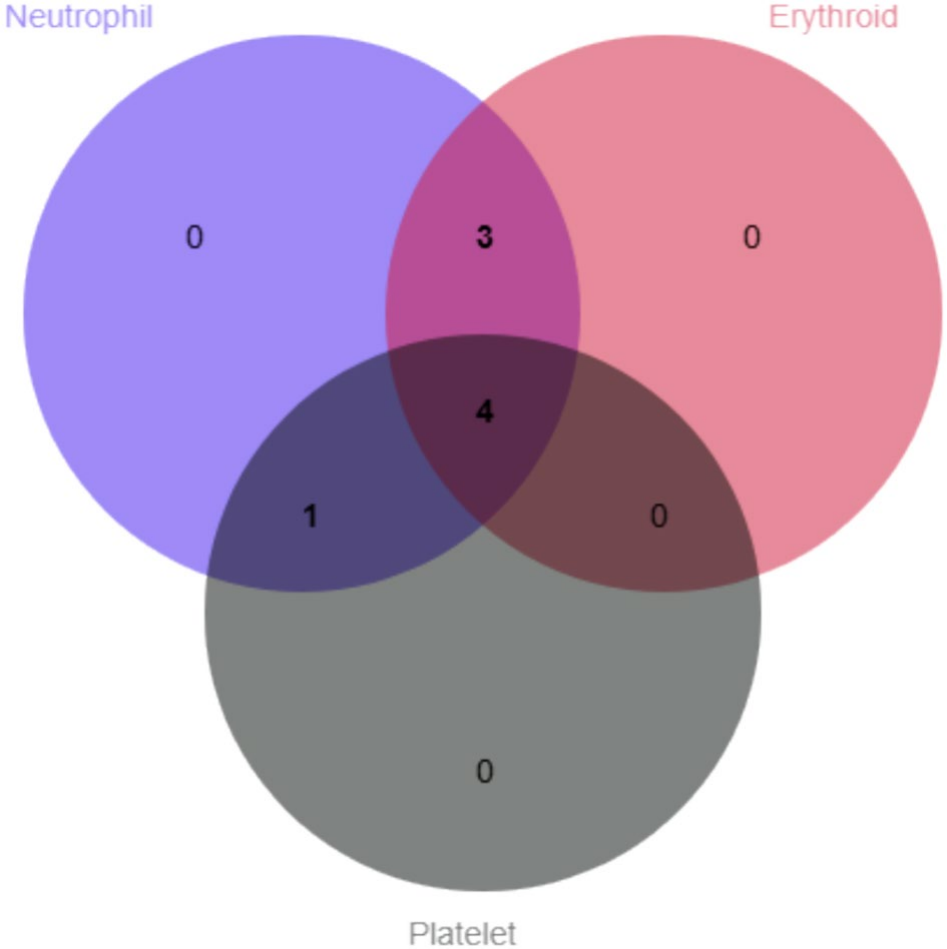
Hematologic responses at 3 months. Venn diagram shows the numbers of patients with single, bilineage and trilineage responses (n = 11).

As shown in Fig. 1, at 3 months post-treatment, 8 patients achieved neutrophil response, 7 attained erythroid response, 5 demonstrated platelet response, 4 achieved trilineage response, while 3 patients showed no response in any lineage.

**Fig. 2:**
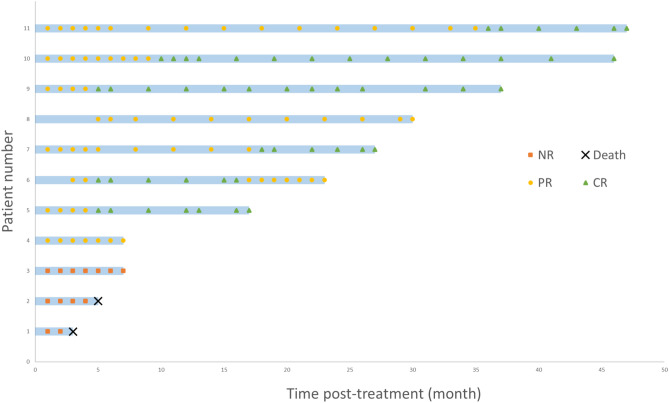
Response status of eleven AA patients shown in swimmer plots. Patients underwent laboratory testing at least monthly until remission was achieved, with subsequent testing frequency reduced based on clinical requirements.

At the 6-month post-treatment evaluation, eight patients achieved trilineage response while the remaining three showed no response. As shown in Fig. 2, among the eight trilineage responders, five achieved sustained CR, one maintained CR for 1 year before declining to PR, and two attained PR. The overall response rate was 8/11, with a CR rate of 5/11.

Trilineage response: the median time to achieve trilineage response in eight patients was 112 days (range: 18–168 days) post-treatment. At the end of follow-up, the median duration of sustained trilineage response was 24 months (range: 6–44 months).

Granulocytic response: four patients with baseline ANC > 1.5 × 10⁹/L maintained normal granulocyte counts, while four others achieved granulocytic response with a median time of 19 days (range: 8–60 days) post-treatment. The median duration of granulocytic response was 38 months (range: 6–45 months).

Erythroid response: one patient had a baseline hemoglobin level > 100 g/L. Seven patients achieved erythroid response with a median time of 29 days (range: 8–161 days) post-treatment, and the median duration of erythroid response was 30 months (range: 6–46 months).

Platelet response: the median time to platelet response in eight patients was 89 days (range: 18–168 days) post-treatment, with a median duration of 24 months (range: 6–44 months).

### Survival

The median follow-up time was 23 months (range: 2–47 months), with 2 deaths and 9 survivors. Both fatalities occurred in patients with SAA. The first was a 74-year-old male who failed to achieve response and died from pulmonary hemorrhage 3 months post-treatment. The second was a 70-year-old male with no response who succumbed to pulmonary infection 5 months after treatment.

### Clonal evolution

At enrollment, one patient exhibited a + 8 chromosomal abnormality, while another harbored MDS-associated STAT3 (VAF, 2.93%) and STAT5B (VAF, 1.26%) gene mutations. During follow-up, laboratory tests and bone marrow smears revealed no evidence of progression to MDS or AML, with genetic testing not repeated due to financial constraints.

### Safety

All patients tolerated cord blood well, with no allergic or toxic reactions observed and no clinical occurrence of acute or chronic GVHD. For the survivors, no adverse events necessitating drug discontinuation occurred.

## Discussion

Currently, the combination of ATG, cyclosporine, and eltrombopag (ACE regimen) is recognized as the most effective IST for severe aplastic anemia. Two prospective clinical trials led by the NIH and EBMT^23,24^ demonstrated improved response rates with the ACE regimen, achieving 6-month CR rates of 58% and 32%, respectively. However, a substantial proportion of patients remain refractory to or relapse after IST, or cannot receive ATG due to adverse effects. Therefore, for those ineligible for allogeneic hematopoietic stem cell transplantation owing to age or donor availability constraints, therapeutic options remain limited.

In our study, a regimen combining cyclosporine and hetrombopag, with cord blood infusion serving as an immunomodulator, was applied in the treatment of AA. For SAA, the total response rate was 4/6, including 3 CRs, comparable to reported outcomes of the ACE regimen^23,24^. In non-severe cases, the response rate was 4/5, with 2 CRs, aligning with published rates for cyclosporine plus hetrombopag in non-severe aplastic anemia (29% CR rate, 93% overall response rate)^25^. The overall response rate was comparable to that achieved with the hetrombopag plus IST regimen^26–28^, while avoiding adverse events associated with ATG. Although hematopoietic stem cell transplantation represents a curative option for young patients with SAA, with 5-year survival rates exceeding 80%^1^, all transplant-eligible patients in this study declined the procedure due to perceived risks and financial constraints.

As documented in previous studies, advanced age constitutes an adverse prognostic factor in aplastic anemia patients^29^. Elderly patients also exhibited poorer treatment responses^30^. In the present study, all three non-responding patients were over 60 years of age, with two succumbing within six months post-treatment. This suboptimal therapeutic response may be attributed to the frailty and compromised organ function in elderly individuals, which heightens susceptibility to hemorrhagic events and severe infections. Furthermore, age-related decline in bone marrow regenerative capacity likely impedes favorable response to therapy.

Therefore, this study represents a preliminary exploration of a novel treatment regimen for refractory/relapsed AA patients and those ineligible for ATG therapy. In this protocol, cyclosporine delivers immunosuppressive effects. While the therapeutic rationale for hetrombopag remains partially unclear, though TPO-RAs have been proposed to stimulate hematopoiesis via TPO receptors on early progenitor cells^31^—analogous to endogenous TPO’s role in regulating and maintaining hematopoietic stem cells^32^—this theory conflicts with the naturally elevated TPO levels observed in aplastic anemia patients^33^. Similar to eltrombopag, hetrombopag’s efficacy may stem from multiple mechanisms^34^: immunomodulatory activity, promotion of transforming growth factor-beta (TGF-β) secretion, inhibition of dendritic cell differentiation, suppression of pro-inflammatory cytokine release, and iron-chelating properties^35^.

The immunomodulatory properties of cord blood^15^, its established role in enhancing hematopoietic reconstitution during stem cell transplantation^16,17^, and therapeutic applications in autoimmune diseases^21^ prompted its inclusion in our AA treatment strategy. Cord blood cellular components may modulate the bone marrow microenvironment and promote hematopoietic stem/progenitor cell expansion^36^, while avoiding the toxic effects associated with ATG. Among 28 infusions, none of the 11 patients exhibited allergic reactions, toxicity, or GVHD. These attributes render this approach particularly suitable for initial treatment in elderly or debilitated patients. However, the lack of a control arm makes it difficult to attribute observed responses specifically to cord blood.

Beyond hetrombopag and eltrombopag, combination therapies with other TPO-RAs may represent novel alternatives. Prospective clinical trials have demonstrated the efficacy and favorable tolerability of romiplostim monotherapy in relapsed aplastic anemia patients^37–39^, while retrospective studies indicate that high-dose romiplostim (20 μg/kg) can be effective in eltrombopag-refractory relapsed cases^40^. Furthermore, retrospective analyses of avatrombopag monotherapy in refractory/relapsed patients reveal comparable efficacy to eltrombopag^41^. For non-responders in our study, switching to alternative TPO-RAs may be beneficial.

In conclusion, this small-scale study introduces a novel regimen combining umbilical cord blood infusion, cyclosporine, and hetrombopag for treating aplastic anemia. With a median follow-up of 23 months, the overall response rate reached 8/11 and the CR rate 5/11. The safety and efficacy of this regimen were preliminarily assessed, suggesting a potential therapeutic alternative for patients ineligible for standard immunosuppressive therapy.

## Data Availability

The data analyzed in the current study are available from the corresponding author on reasonable request.
